# Supplementary material for: Biochar-Mediated Zirconium Ferrite Nanocomposites for Tartrazine Dye Removal from Textile Wastewater
Source: Nanomaterials (Basel). 2022 Aug 17;12(16):2828. doi: 10.3390/nano12162828 (PMC9414429; doi:10.3390/nano12162828)
Supplement: Supplementary file 1 [file nanomaterials-12-02828-s001.zip › nanomaterials-1841674-supplementary.pdf]

nanomaterials

Article

Biochar-Mediated Zirconium Ferrite Nanocomposites for Tartrazine Dye Removal from Textile Wastewater

Shazia Perveen [1], Raziya Nadeem [1], Farhat Nosheen [2], Muhammad Imran Asjad [3], Jan Awrejcewicz [4] and Tauseef Anwar [5,*]

1 Department of Chemistry, University of Agriculture, Faisalabad 38000, Pakistan
2 Institute of Chemistry, University of Sargodha, Sargodha 40100, Pakistan
3 Department of Mathematics, University of Management and Technology, Lahore 54000, Pakistan
4 Department of Automation, Biomechanics and Mechatronics, Lodz University of Technology, Żeromskiego 116, 90-924 Łódź, Poland
5 Department of Physics, The University of Lahore, Lahore 54000, Pakistan
* Correspondence: tauseef.anwar@phys.uol.edu.pk

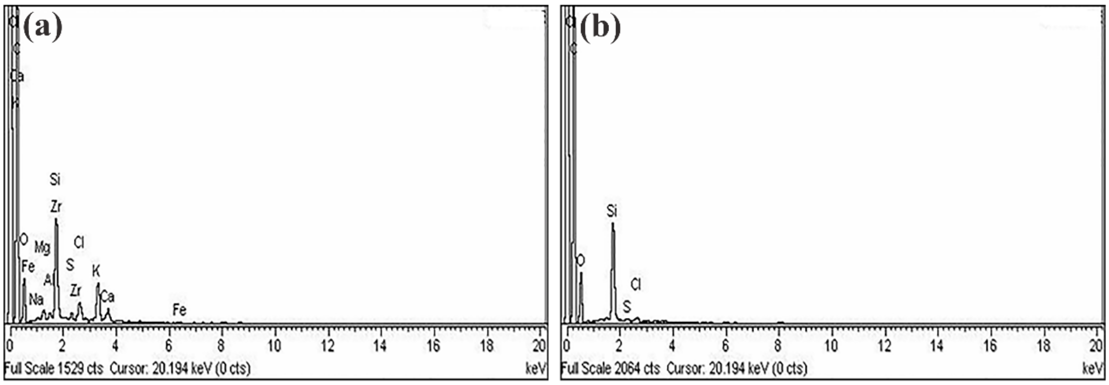

Figure S1. EDX spectra of (**a**) BC-$ZrFe_2O_5$ NCs and (**b**) dye loaded BC-$ZrFe_2O_5$ NCs.

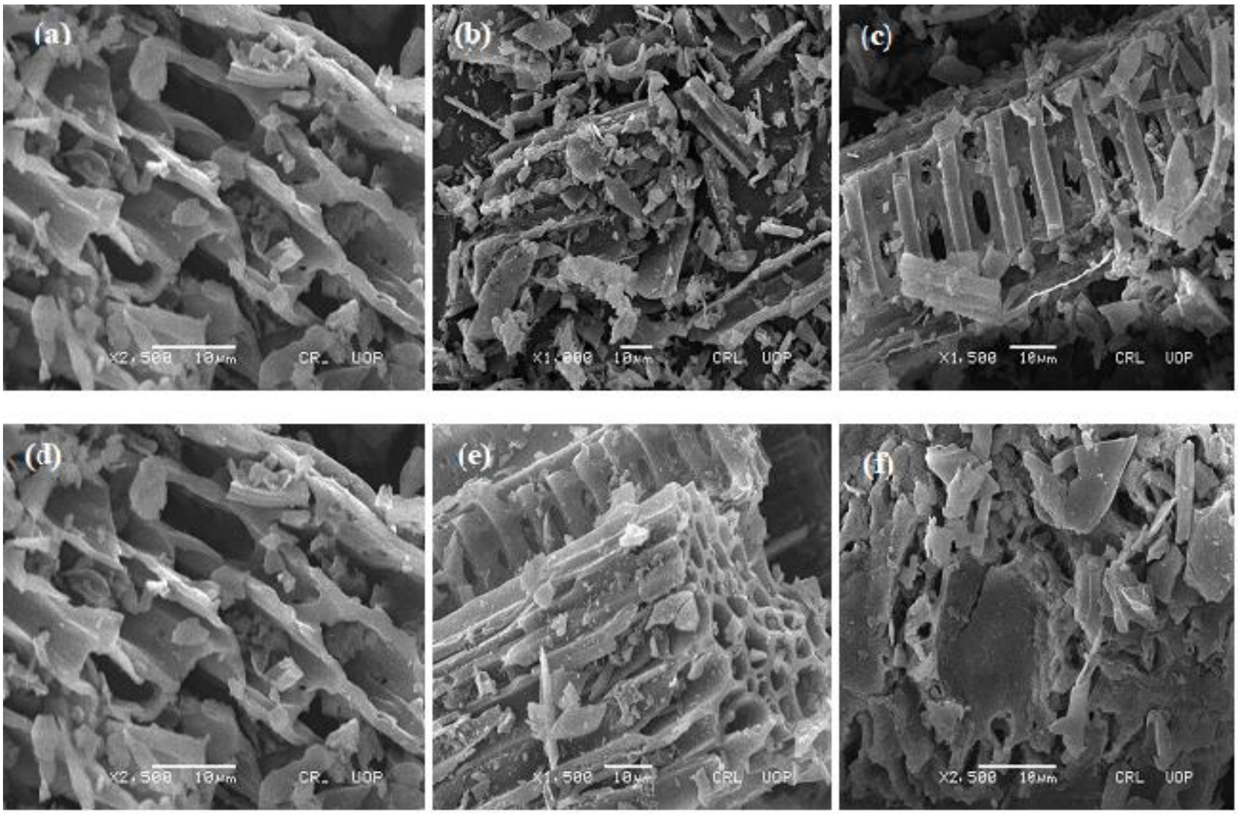

Figure S2. SEM micrographs of WSBC (**a**,**b**,**c**) and Tartrazine dye loaded WSBC (**d**,**e**,**f**) at three different magnification levels.

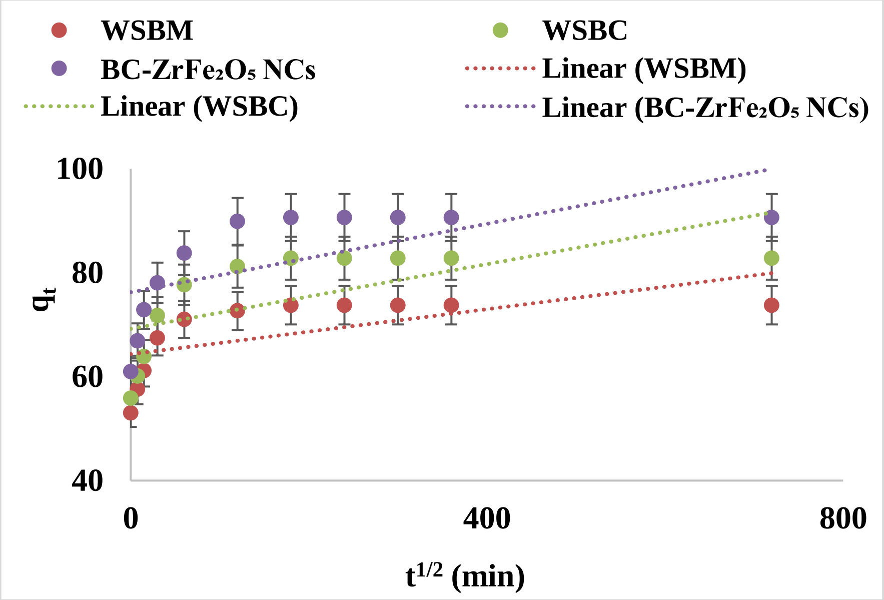

Figure S3. Intraparticles diffusion adsorption kinetic model plot for adsorption of Tartrazine dye by WSBM, WSBC, and BC-$ZrFe_2O_5$ NCs.

Table S1. ANOVA results for Response Surface Quadratic Model of Tartrazine dye using BC-$ZrFe_2O_5$ NCs.

Source	*Sum of Square*	*Df*	*Mean Square*	*F Value*	*p-value Prob > F*	
**Model**	14021.71	14	1001.2	18.52	< 0.0001	Significant
**A-pH**	19.28	1	19.28	0.23	< 0.0001	
**B-Conc.**	13796.35	1	13796.35	164.04	< 0.0001	
**C-Dose**	2.80	1	2.80	0.033	< 0.0001	
**D-Time**	12160.33	1	12160.33	61	< 0.0001	
**AB**	0.81	1	0.81	9.289	< 0.0001	
**AC**	1.62	1	1.62	0.018	< 0.0001	
**AD**	20.25	1	20.25	0.10	< 0.001	
**BC**	1.53	1	1.53	0.018	< 0.005	
**BD**	3969	1	3969	20.01	< 0.0005	
**CD**	6.25	1	6.25	0.32	< 0.05	
**A^2**	1.42	1	1.42	0.017	0.0993	
**B^2**	194.52	1	194.52	2.31	0.0593	
**C^2**	0.68	1	0.68	8.053	0.0303	
**D^2**	6555.96	1	6555.96	33.05	< 0.0001	
**Residual**	841.06	14	84.11			
**Lack of Fit**	841.06	10	168.21			non-significant
**Pure Error**	0.000	4	0.000			
**Cor Total**	14862.77	28				
**Std.Dev.**	3.25			**R^2**	0.9976	
**C.V.**	4.28			**Pred. R^2**	0.9812	
**Mean**	46.98			**Adj. R^2**	0.9953	
**Adequate precision**	32.45			**Press**	723	

Table S2. Comparison between pseudo-first order, pseudo-second-order and intraparticles diffusion kinetic models for Tartrazine Dye.

		**Pseudo first order**			**Pseudo second order**				**Intraparticle Diffusion**		
**Sorbate (Dye)**	**Sorbent**	**q_e (mg g^{-1})**	**K_1ad (min^{-1})**	**R^2**	**q_{exp} (mg g^{-1})**	**q_e (mg g^{-1})**	**K_2ad (g mg^{-1} min^{-1})**	**R^2**	**K_{pi} (mg/g $min^{1/2}$)**	**C_i**	**R^2**
**Tartrazine**	**WSBM**	0.706	−0.004	0.610	66.29	53.64	0.00289	0.997	0.0148	−0.213	0.996
	**WSBC**	0.058	−0.001	0.132	73.74	79.49	0.00056	0.998	0.013	−0.164	0.996
	**BC-$ZrFe_2O_5$ NCs**	8.496	−0.001	0.533	82.811	89.22	−0.0005	0.999	0.011	0.058	0.989

Table S3. Equilibrium isotherm parameters for Tartrazine Dye.

Sorbate (Dye)	Sorbent	Langmuir model X_m (mg g^{-1})	K_L (L mg^{-1})	R^2	q_{exp} (mg g^{-1})	Freundlich model q_e (mg g^{-1})	1/n	K_F (mg g^{-1})	R^2
Tartrazine	WSBM	111.12	0.060	0.8671	66.29	53.64	0.49	0.077	0.9895
	WSBC	144.93	0.055	0.8608	73.74	79.47	0.63	0.025	0.9899
	BC-$ZrFe_2O_5$ NCs	232.56	0.039	0.7174	83.81	89.22	0.77	0.012	0.9907
